# Synthesis and Characterization of K and Eu Binary Phosphides

**DOI:** 10.3390/ma12020251

**Published:** 2019-01-13

**Authors:** Juli-Anna Dolyniuk, Justin Mark, Shannon Lee, Nhon Tran, Kirill Kovnir

**Affiliations:** 1 Department of Chemistry, University of California, Davis, CA 95616, USA; jdolyniukjohnson@gmail.com (J.-A.D.); tran.r15@gmail.com (N.T.); 2 Department of Chemistry, Iowa State University, Ames, IA 50011, USA; jmark@iastate.edu (J.M.); shelee@iastate.edu (S.L.); 3 Ames Laboratory, U.S. Department of Energy, Ames, IA 50011, USA

**Keywords:** Zintl phases, polyphosphides, synthesis, crystal structures, optical properties

## Abstract

The synthesis, structural characterization, and optical properties of the binary Zintl phases of *α*-EuP_3_, *β*-EuP_3_, EuP_2_, and *α*-K_4_P_6_ are reported in this study. These crystal structures demonstrate the versatility of P fragments with dimensionality varying from 0D (P_6_ rings in *α*-K_4_P_6_) to 1D chains (EuP_2_) to 2D layers (both EuP_3_). EuP_2_ is isostructural to previously reported SrP_2_ and BaP_2_ compounds. The thermal stabilities of the EuP_2_ and both EuP_3_ phases were determined using differential scanning calorimetry (DSC), with melting temperatures of 1086 K for the diphosphide and 1143 K for the triphosphides. Diffuse reflectance spectroscopy indicated that EuP_2_ is an indirect semiconductor with a direct bandgap of 1.12(5) eV and a smaller indirect one, less than 1 eV. Both EuP_3_ compounds had bandgaps smaller than 1 eV.

## 1. Introduction

An impressive variety of polyphosphides exist in the solid state [1,2,3,4,5,6,7,8,9,10,11,12,13,14,15,16,17,18,19,20,21,22,23,24,25,26,27,28,29,30,31,32,33]. In these systems, P atoms form various coordination environments and multidimensional P fragments with a broad array of *s*, *p*-, and *f*-elements. On the basis of a 1988 review on polyphosphides, nearly a dozen binary phases exist in the K-P system, and only slightly fewer Eu–P binary phases exist [1,2]. Due to the presence of a partially filled *f*-electron shell on Eu atoms, some Eu phosphides show interesting magnetic properties [34,35].

The P fragments in these systems range from 0D to 3D, involving isolated atoms, dumbbells, rings, clusters, chains, layers, and frameworks of P atoms. *A*_4_P_6_ (*A* = K, Rb, Cs) compounds have a planar cyclic P structural unit, hexaphosphabenzene (P_6_^4–^), of the point group *D*_6h_ (6/*mmm*). A similar As_6_^4−^ anion was also reported [5,36,37,38]. 

All of these compounds belong to a broad family of Zintl phases [39,40]. The electropositive cations donate their valence electrons to the P anions, realizing a stable electron configuration of the noble gas. Phosphorus polyanions accept electrons and achieve an electron octet by forming P–P bonds and/or by forming electron lone pairs. In such a formalism, isolated P atoms bear a −3 charge, P atoms forming P–P dumbbells each have a −2 charge, while P atoms forming two and three homoatomic P–P bonds have −1 and 0 charges, correspondingly.

Most of alkali- or alkaline-earth phosphides are expected to be semiconductors on the basis of their charge-balanced nature. The bandgaps of these systems vary, leading to a range of colors from yellow to red and gray or black [1,6,7]. Recent discoveries in metal polyphosphide systems expand upon this library, introducing unique polyphosphide fragments which may be different from any previously reported [6,7]. Furthermore, an evaluation of the inorganic crystal structure database reveals that our understanding of many of the published binary phosphides is incomplete. In the current work, we present crystal structure information for some reported but only partially characterized or uncharacterized binary phosphides: *α*-EuP_3_, *β*-EuP_3_, EuP_2_, and *α*-K_4_P_6_.

## 2. Materials and Methods 

### 2.1. Synthesis 

All manipulations with the initial materials and sample handling were performed inside an Ar-filled glove box (*p*(O_2_) < 1 ppm). The starting materials of metallic europium (U.S. DOE Ames Laboratory, 99.9%), metallic potassium (Alfa Aesar, 99.95%), and red phosphorus (Alfa Aesar, 99%) were used as received.

Single crystals of *β*-EuP_3_ were originally synthesized as a side-product of the synthetic endeavors in the Eu-Ni-P system. The reactants (total amounts 500 mg) were placed in a glassy carbon crucible and sealed in a silica tube under vacuum; the residual pressure was 0.04 mbar. The annealing profile involved the ramping of reactants up to 1073 K over 17 h and holding at the maximum temperature for 140 h. The resulting products were gray-metallic binary Ni-phosphides and darker, blackish crystals of *β*-EuP_3_. Likewise, the original single crystals of *α*-EuP_3_ were found as a minor side product of a reaction of elemental Eu and P in a 1:3 stoichiometry sealed in a carbonized silica tube under vacuum. The sample was ramped up to 1173 K over 17 h and annealed for 48 h. Subsequent syntheses to form *α*-EuP_3_ samples were similar, but the samples were held at 1123 K for 217 h. *β*-EuP_3_ was formed by quenching from the melt at 1123 K. Alternatively, *α*-EuP_3_ was formed by controlled cooling of the melt over the course of 5 h. 

EuP_2_ crystals were originally obtained by the low temperature attempt to make EuP_3_ from elements at 973 K. Eu and P were combined in a 1:3 stoichiometry in a carbonized silica ampoule and sealed under vacuum. The sample temperature was gradually increased to 973 K over 17 h and held there for 48 h. The reaction was incomplete after a single heating, where EuP_2_ formed on the surface of the partially reacted Eu. Therefore, to improve the yield of EuP_2_, the sample required additional grinding and re-annealing under the same conditions. 

A single crystal of *α*-K_4_P_6_ was originally synthesized as a product of the reaction of elemental K with P in a 1:1.1 stoichiometry in an attempt to make the stoichiometric phase KP. Elemental P was placed at the bottom of a silica tube, and K was scooped into a small alumina crucible. The crucible was subsequently placed gently into the silica tube on top of the P, and the tube was sealed under vacuum. The sample was ramped up to 773 K over 17 h and annealed for 24 h. The resulting blackish product was found outside the crucible, in the bottom of the silica tube.

### 2.2. Single-Crystal X-ray Diffraction

A part of sample was placed in a Petri dish and covered with Paratone oil in the Ar-filled glove box (Hampton research corp, USA). Single crystals were selected using an optical microscope and placed under a stream of cold dry nitrogen on the single-crystal diffractometer. Datasets were collected on either a Bruker Apex II diffractometer or a Bruker D8 Venture diffractometer, both using Mo-*K*α radiation. Data for *α*-EuP_3_, *β*-EuP_3_, and *α*-K_4_P_6_ were collected at 0.5° step width and 1 s per frame exposure time, while data for EuP_2_ were collected at 0.5° step width with a collection time of 10 s per frame. Data collection and integration and space group determination were performed using a Bruker APEX3 program suite (Bruker AXS USA). Multiscan absorption correction was applied to all crystals. Important single-crystal refinement parameters of all phases can be found in Table 1. Several crystals of EuP_2_ were measured, all exhibiting similar not-very-high qualities, presumably due to partial decomposition. The solutions and refinements of crystal structures were carried out using the SHELX suite of programs [41]. Further details of the crystal structure determinations may be found through Cambridge Crystallographic Data Centre by using CCDC #1885037-1885040 or in the Supplementary Materials.

### 2.3. X-ray Powder Diffraction

**Figure 1:**
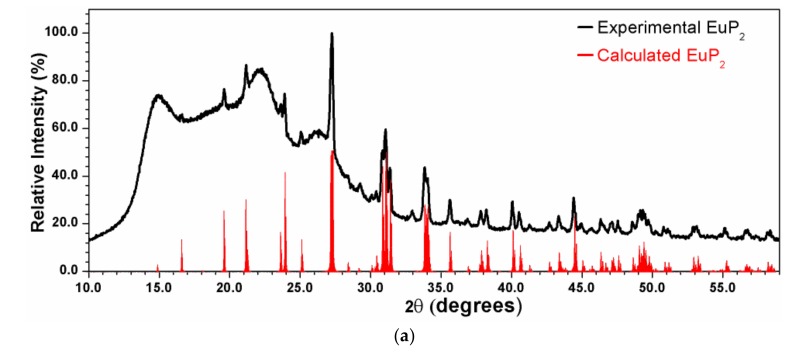

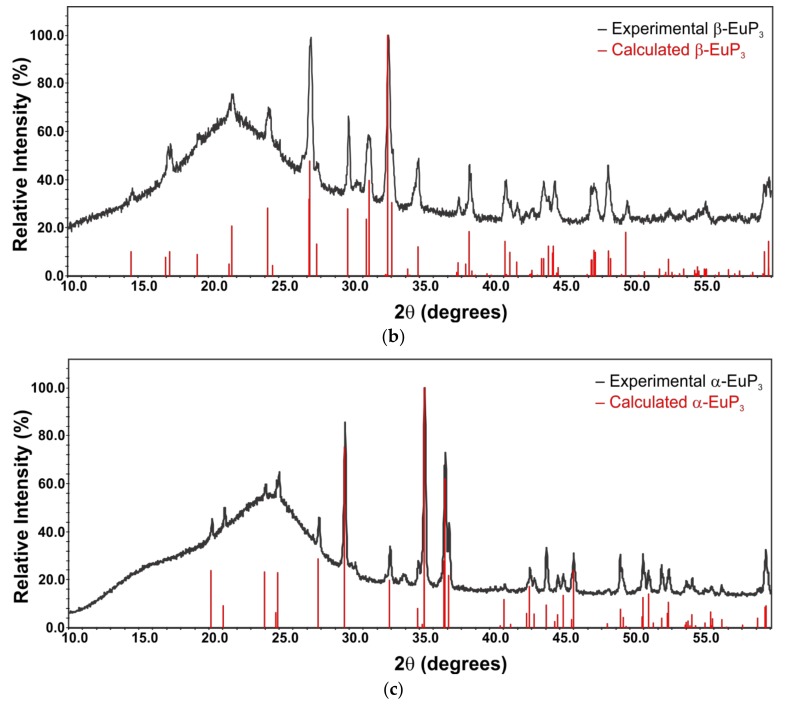
X-ray powder diffraction patterns for EuP_2_ (**a**) and EuP_3_ (**b** and **c**) samples along with their calculated patterns from single-crystal data. The high background in the two bottom patterns may be due to the unidentified amorphous admixtures occurring as a result of partial sample decomposition in air. While the target phosphide is the main phase, low-intensity diffraction peaks of unidentified admixtures are present.

X-ray powder diffraction patterns were measured using a Rigaku Miniflex 600 with Cu-*K*α radiation and a Ni-*K*_β_ filter. The experimental patterns were compared with the calculated ones based on the crystal structure models from single-crystal X-ray diffraction experiments. EuP_3_ phases were stable in air for a short period of time, which was sufficient for the purpose of X-ray powder diffraction (<10 min exposure to air). However, the quick decomposition of EuP_2_ and K_4_P_6_ in air at ambient temperature was evident by abrupt changes in color. Thus, homemade Kapton air-free holders were used for X-ray powder diffraction of EuP_2_ (Figure 1a).

### 2.4. Differential Scanning Calorimetry

Two Netzsch Differential Scanning Calorimeters (DSC), either Netzsch STA 449 F3 Jupiter or Netzsch DSC 404 F3 Pegasus, were used to characterize the thermal behavior of synthesized phases. In order to maintain similar conditions to those of actual syntheses, DSC measurements were run using small evacuated and sealed silica ampoules with enough sample to cover the base of the ampoule (approximately 30–50 mg). The samples were initially heated to 673 K at a rate of 10 K/min, then the heating was slowed to 5 K/min over the 673–1273 K range. A similar cooling scheme was employed. Errors in the melting and crystallization temperatures were estimated not to exceed +/−3 K.

### 2.5. Solid-State Diffuse Reflectance Spectroscopy

Solid-state UV–Vis spectroscopy (Thermo Scientific Evolution 220 Spectrometer or BLACK-Comet C-SR-100 spectrometer, manufacturer, city, country) was employed for experimental bandgap determinations. Kubelka–Munk diffuse reflectance was used to help characterize the bandgaps of the three Eu-containing phases: *α*-EuP_3_, *β*-EuP_3_, and EuP_2_. For UV–Vis diffuse reflectance measurements, solid samples were ground into powders and heat-sealed in transparent polyethylene bags inside an Ar-filled glove box to prevent any phase degradation or color changes. A blank measurement of an empty polyethylene bag was used as a reference.

## 3. Results and Discussion

### 3.1. Synthesis and Thermal Stability

All synthesized polyphosphide phases decomposed in air over time. K_4_P_6_ was much more air-sensitive than the Eu phases. Even in paratone oil, the rich black and gold-tinted blocks of K_4_P_6_ fizzled and bubbled, turning a light red-orange color. The reactivity of K with silica and its high vapor pressure made it very difficult to control the amount of K in the reaction, thus K_4_P_6_ was formed in an excess of K. Furthermore, a strong reaction of K with the silica ampoule was observed by the discoloration of the ampoule’s inner surface. Thus, only single crystals of K_4_P_6_ were obtained, but no pure-phase synthesis was managed. The synthesis and thermal decomposition of *α*-K_4_P_6_ were previously explored by von Schnering et al. [5]. Their synthesis involved a stoichiometric reaction of elemental K with P up to a maximum temperature of 870 K. On the basis of their results, the selective synthesis of the *α*-phase requires the slow cooling of the K_4_P_6_ melt. Additionally, they showed the *α*-K_4_P_6_ phase decomposes into a K-deficient phase of K_3_P_7_ starting at 650 K (7K_4_P_6_ → 6K_3_P_7_ + 10K), which then begins to sublime at ≈ 830 K [5]. Because of the reactivity of *α*-K_4_P_6_ in air and lack of phase purity, the thermal stability of this phase was not further explored.

EuP_2_ was also air-sensitive, though less than K_4_P_6_. While the latter decomposed readily in oil, EuP_2_ did not. However, the phase decomposed when left in air, changing color to a light orange within minutes. EuP_2_ was synthesized from a 1:3 ratio of Eu/P at 973 K. Powder X-ray diffraction on initial annealed samples revealed little EuP_2_ formation; however, grinding and re-annealing this sample a second time drastically improved the yield of EuP_2_. The phosphorus excess sublimed to the top of the ampoule.

The syntheses of EuP_3_ phases were more straightforward. Oxidized portions of Eu were removed from the elemental metal, and the reaction of Eu with P at a low temperature (1073 K) formed a high-yield sample of *β*-EuP_3_. Tiny admixtures of *α*-EuP_3_ were discovered as a minor product. In addition to the formation of EuP_2_, work by von Schnering et al. in the early 1980s showed the possibility of forming *β*-EuP_3_ via the decomposition of EuP_7_ [3]. Subsequent syntheses to form EuP_3_ samples were conducted at a higher temperature, 1123 K. β-EuP_3_ was formed by quenching the sample at 1123 K. Alternatively, α-EuP_3_ was formed by controlled cooling of the sample over the course of 5 h. Typical X-ray powder diffraction patterns are shown in Figure 1.

**Figure 2:**
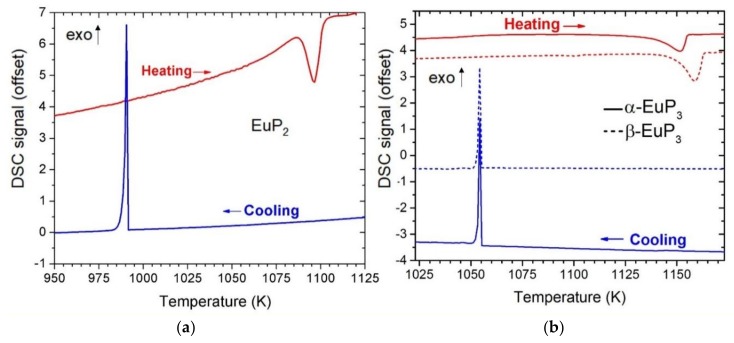
DSC curves for EuP_2_ (**a**) and EuP_3_ (**b**) phases. Heating is shown in red, and cooling is shown in blue, exothermic direction is shown with exo arrow. For EuP_3_, the solid lines represent the *α*-phase, and the dashed lines represent the *β*-phase.

Differential scanning calorimetry (DSC) was used to characterize the thermal stability of the Eu-containing compounds (Figure 2). Based on these results, both EuP_3_ phases had similar thermal stability melting and recrystallizing at around 1143 K and 1055 K, respectively. Previous results have shown *α* and *β* phases of binary phosphides may have very similar melting/decomposition temperatures. For example, in the case of dimorphic BaP_3_ phases, the melting temperatures were within error of one another at 1105(3) K and 1109(3) K for *mP*-BaP_3_ and *mS*-BaP_3_, respectively [6]. *α*-EuP_3_ has a 35 K higher melting point than the isostructural analogue *mS*-BaP_3_. EuP_2_ was found to melt at approximately 1086 K and recrystallize at 991 K. Powder diffraction on the DSC sample of EuP_2_ confirmed the recrystallization to be that of EuP_2_. This melting point is between those reported for BaP_2_ (1053 K) and SrP_2_ (1123 K) [7].

### 3.2. Crystal Structures

**Figure 3:**
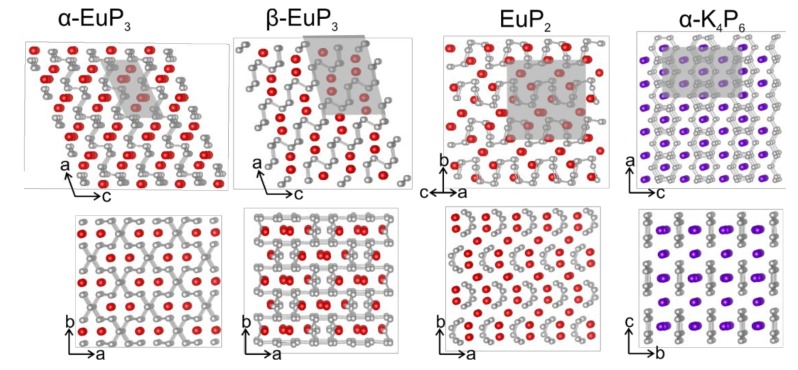
The crystal structures of two EuP_3_ phases, EuP_2_, and *α*-K_4_P_6_ are shown along two different directions. The unit cells are highlighted with gray boxes. P: gray, Eu: red; K: purple.

The crystal structures of *α*-EuP_3_ [1], *β*-EuP_3_ [1,34,35], EuP_2_ [1,3], and *α*-K_4_P_6_ [1,5] were mentioned in earlier works, although their complete crystal structure information is not available. For *α*-K_4_P_6_ and *β*-EuP_3_ the crystal structures determined by us agree well with previous model based on X-ray powder and single-crystal neutron diffraction data [5,35]. For *α*-EuP_3_ and EuP_2_, the structural models were proposed based on similarities of the unit cell parameters and powder diffraction patterns [1,3]. We confirmed the proposed models. The crystal structures of *α*- and *β*-EuP_3_ belong to well-known structure types in the realm of electropositive metal polyphosphides. The former is isostructural to *mS*-BaP_3_, and the latter is isostructural to SrP_3_. Both phases are made up of layers of infinite P sheets with Eu atoms sitting between the layers. Both P layers can be related to the layers of black P (Figure 3). Though both phases look nearly identical down the [010] direction, the ordering of their layers is different.

**Figure 4:**
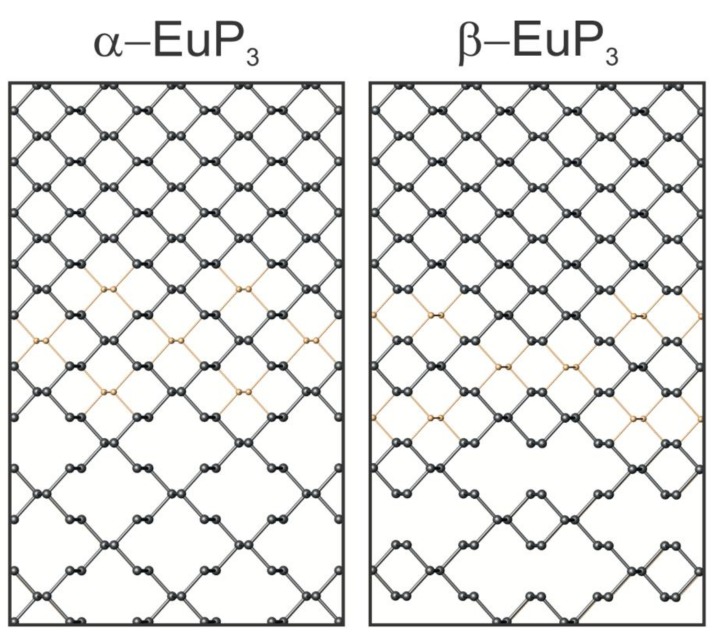
The evolution of phosphorus frameworks from black phosphorus (top) to the two phases of EuP_3_ (bottom) are shown. The gold atoms depicted (middle) indicate the P atoms that are absent in the EuP_3_ frameworks.

In the case of *α*-EuP_3_, 14-membered P rings are present in the infinite P layers. These rings can be formed directly from black P by the removal of alternating P–P dumbbells in two directions. Interestingly, the infinite chair-like puckering in these P layers is maintained upon the evolution of P_3_^2-^ layers from black P (Figure 4). Similarly, two pairs of neighboring P–P dumbbells from the black P framework can be removed to form the 22-membered P rings in *β*-EuP_3_. These large rings are separated by leftover 6-membered P rings from the black P framework. Similar to *α*-EuP_3_, the black P puckering of the layers is maintained in *β*-EuP_3_ (Figure 4). In both EuP_3_ crystal structures, there are two-bonded (2*b*-) and three-bonded (3*b*-) P atoms in a 2:1 ratio. 3*b*-P atoms covalently connected to three P atoms have a formal oxidation state of 0, similar to P atoms in black phosphorus. 2*b*-P atoms, which are connected to only two P atoms, have a formal oxidation state of −1, thus making the compounds electron-balanced: (Eu^2+^)(2*b*-P^1−^)_2_(3*b*-P^0^)_1_. 

EuP_2_ is isostructural to SrP_2_ and BaP_2_ [7]. As opposed to two-dimensional layers in the EuP_3_ phases, the P fragments of EuP_2_ consist of one-dimensional twisted chains of P (Figure 3). The unit cell parameters for EuP_2_ are slightly smaller than those of SrP_2_ [7]. A similar decrease in the unit cell parameters was observed in the crystal structures of *A*_2_SiP_4_ compounds, *A* = Sr, Eu [19].

*α*-K_4_P_6_ is very different from the other three phases, which all show some type of puckered infinite P fragments. The P in K_4_P_6_ is present as isolated 6-membered P_6_^4-^ rings, hexaphosphabenzenes, which can be derived from planar graphene-like P-layers by an ordered removal of a ¼ of P atoms [5]. The rings line up, but are not bound to each other, forming nearly flat layers of isolated hexagons, and the K atoms sit between the layers. The rings are planar, with *D*_6h_ symmetry (6/*mmm*), likely due to their coordination with K atoms that coordinate to the faces of the rings. Previous works have explored these unique P rings in both the solid state and in solution, and As_6_^4−^ analogues are also known to exist [36,37,38].

**Figure 5:**
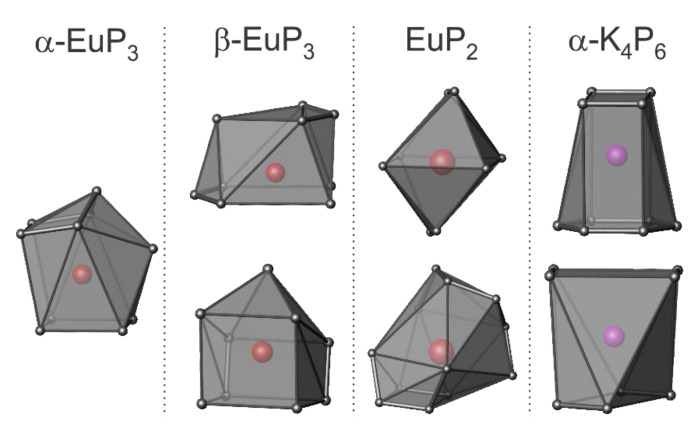
The cations’ coordination in EuP_3_ phases, EuP_2_, and α-K_4_P_6_. P atoms are shown in gray, Eu atoms are red, and K atoms are purple.

The coordination spheres of Eu and K in the four phases are shown in Figure 5. *α*-EuP_3_ has one unique Eu site, while the other three phases have two unique Eu or K sites. Of the three Eu phases, the shortest Eu–Eu distance is present in *β*-EuP_3_ at 3.83 Å. The shortest K–K distance in *α*-K_4_P_6_ is 4.14 Å. Between the Eu and K samples, the P–P distances vary significantly. The shortest P–P distance for *α*-K_4_P_6_ is 2.152(1) Å, while the shortest P–P in Eu-containing compounds is 2.183(5) Å in EuP_2_. The former short length is close to the expected bond lengths in the P_6_^4−^ rings based on other *A*_4_P_6_ systems where *A* = Rb, Cs. This is due to the partial increase of the bond order over one by the formation of a quasi-aromatic π-system. Alternatively, the shortest length in EuP_2_, 2.183(5) Å, is close to the distance of a single P–P bond in black P at 2.23 Å at 300 K [1]. Furthermore, the closest distances between the P “layers” of each phase are 3.32 Å, 3.21 Å, 4.21 Å, and 4.68 Å for *α*-EuP_3_, *β*-EuP_3_, EuP_2_, and *α*-K_4_P_6_, respectively. The increased distances between the “layers” of P chains in EuP_2_ with respect to EuP_3_ phases can be explained by the higher ratio of Eu atoms to P atoms and the fact that P chains are present instead of layers.

### 3.3. Solid-State Diffuse Reflectance Spectroscopy 

Previously published experimental resistivity measurements have shown semiconducting behavior for *β*-EuP_3_, and, with the structural similarities between the two phases, the *α-*phase may act electronically similar [34,35]. Kubelka-Munk diffuse reflectance was used to determine the bandgaps of the Eu-containing phases. For *α*-EuP_3_ and *β*-EuP_3_, no peaks in the Kubelka-Munk curve were present, indicating the likelihood of small bandgaps of less than 1 eV. This aligns with the observed black colors of the phases. Unfortunately, Tauc plots, and thus bandgap estimations, for the phases would likely be unreliable due to the small size of the bandgaps and the limitations of the instrument.

**Figure 6:**
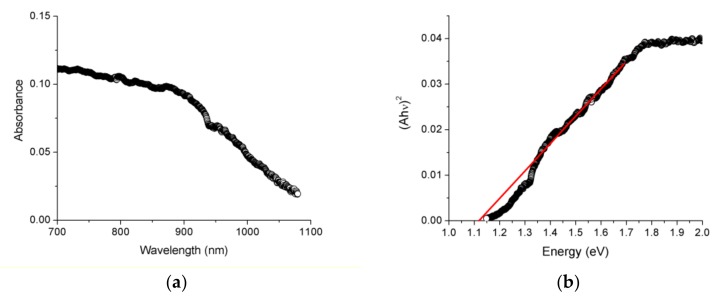
Absorbance spectra (**a**) and solid-state UV-Vis direct bandgap Tauc plot (**b**) for EuP_2_.

In turn, EuP_2_ showed an abrupt increase in absorption (Figure 6). Estimation of the direct bandgap using Tauc plots resulted in the values of 1.12(5) eV. The indirect bandgap appeared to be smaller and cannot be reliably determined because of the limitations of the instrument. The bandgap of EuP_2_ was smaller than the bandgap determined for isostructural SrP_2_, with indirect and direct bandgaps of 1.3(1) eV and 1.5(1) eV, respectively [7].

## 4. Conclusions

Though numerous polyphosphides are known, many have not been fully characterized. It is likely that there may still be other binary phosphides that await discovery. Showing a broad variety of structure types and P fragments, the applications for these materials may be just as broad. Furthermore, a combination of phosphides could lead to interesting intermediates with controlled structures and properties. The four crystal structures of binary phosphide phases introduced here (*α*-EuP_3_, *β*-EuP_3_, EuP_2_, and *α*-K_4_P_6_) are no exception, and they broaden the library of binary polyphosphides. However, the synthesis of these phases is challenging due to the high vapor pressure of phosphorus at the reaction temperatures and the high reactivity and vapor pressure of K metal.

## Figures and Tables

**Table 1:** Data collection and structure refinement parameters.

	*α*-EuP_3_	*β*-EuP_3_	EuP_2_	α-K_4_P_6_
Space Group	*C*2/*m*	*C*2/*m*	*P*2_1_/*c*	*Fmmm*
Temp [K]	100(2)			
*λ* [Å]	Mo *K*α, 0.71073			
*a* [Å]	9.044(1)	11.272(1)	6.1040(8)	8.5508(8)
*b* [Å]	7.2087(9)	7.3390(6)	11.684(2)	9.2899(8)
*c* [Å]	5.5710(7)	8.4242(7)	7.352(1)	14.148(1)
*β* [degree]	113.117(4)	103.304(1)	127.680(5)	
*V* [Å^3^]	334.06(7)	678.2(1)	415.0(1)	1123.9(2)
*Z*	4	8	6	4
*ρ* [g cm^−3^]	4.869	4.796	5.135	2.023
*μ* [mm^−1^]	19.91	19.62	23.45	2.37
*θ* [degree]	3.74 < *θ* < 24.71	2.48 < *θ* < 29.98	3.49 < *θ* < 26.30	2.88 < *θ* < 29.45
data/parameters	259/22	1062/44	844/43	432/18
*R*_1_ (*I* > 2*σ*(*I*))	0.020	0.013	0.042	0.022
*wR*_2_ (*I* > 2*σ*(*I*))	0.041	0.029	0.109	0.033
Goodness-of-fit	1.10	0.96	1.12	1.06
Diff. peak and hole, e/Å^3^	0.91 and −1.13	0.93 and −0.80	3.71 and −2.36	0.48 and −0.47

## Supplementary Materials

Supplementary materials can be accessed at: http://www.mdpi.com/1996-1944/12/2/251/s1, details of the crystal structure determinations.
